# Supplementary material for: Transcriptomic Insights into Mechanisms of Early Seed Maturation in the Garden Pea (Pisum sativum L.)
Source: Cells. 2020 Mar 23;9(3):779. doi: 10.3390/cells9030779 (PMC7140803; doi:10.3390/cells9030779)

Zhewan−1(10) vs Zhon

Global Summary

%DE = 0.88

genes with fdr < 0.2 = 1781 (669 + / 1112 −)

genes with fdr < 0.1 = 1734 (648 + / 1086 −)

genes with fdr < 0.05 = 1651 (596 + / 1055 −)

genes with fdr < 0.01 = 1234 (408 + / 826 −)

genes in genesets = 0

<FC> = 0

<t−score> = −1.77

<p−value> = 0

<fdr> = 0.12

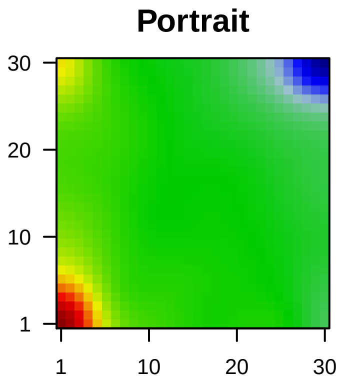

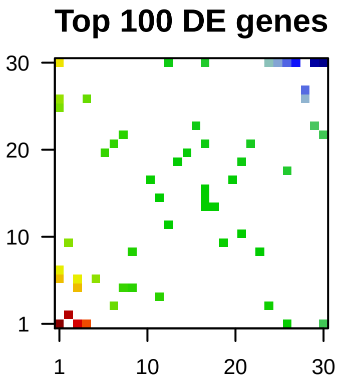

Global Genelist

Rank	ID	log(FC)	p−value	fdr	Metagene	Description
1	TRINITY_DN4	−0.07	1e−10	0.0000008	1 x 5	
2	TRINITY_DN2	1.36	6e−10	0.0000003	1 x 1	
3	TRINITY_DN4	0.04	2e−09	0.0000005	17 x 16	
4	TRINITY_DN1	1.49	4e−09	0.0000005	1 x 1	
5	TRINITY_DN1	8.9	7e−09	0.0000005	1 x 1	
6	TRINITY_DN3	−0.85	9e−09	0.0000002	26 x 30	
7	TRINITY_DN6	1.28	1e−08	0.0000005	1 x 1	
8	TRINITY_DN6	−1.92	1e−08	0.0000003	30 x 1	
9	TRINITY_DN2	2.04	1e−08	0.0000006	1 x 1	
10	TRINITY_DN2	0.03	2e−08	0.0000026	11 x 17	
11	TRINITY_DN5	1.78	4e−08	0.0000026	1 x 30	
12	TRINITY_DN1	−2.39	6e−08	0.0000028	30 x 30	
13	TRINITY_DN4	−0.46	6e−08	0.0000028	27 x 30	
14	TRINITY_DN2	1.73	7e−08	0.0000026	1 x 30	
15	TRINITY_DN1	−1.48	7e−08	0.0000028	30 x 30	
16	TRINITY_DN2	0.89	7e−08	0.0000059	2 x 2	
17	TRINITY_DN1	0.3	1e−07	0.0000062	2 x 10	
18	TRINITY_DN1	0.29	1e−07	0.0000063	3 x 6	
19	TRINITY_DN5	1.79	2e−07	0.0000060	1 x 1	
20	TRINITY_DN1	0.3	2e−07	0.0000060	1 x 7	

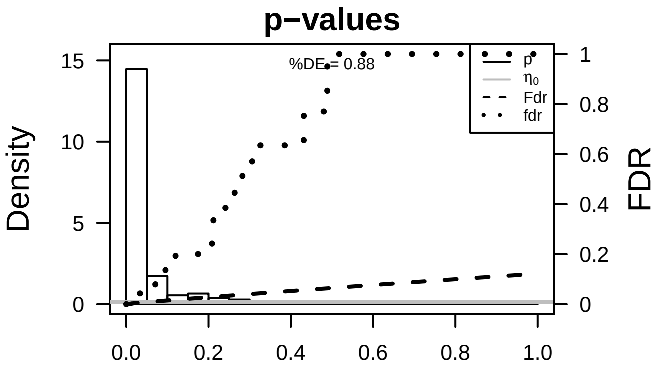

Supplement: Supplementary file 1 [file cells-09-00779-s001.zip › Supplementary materials/FileS4.zip!/FileS3/TFs - Results/Summary Sheets - Differences/Reports/Zhewan.1.10..vs.Zhongwan.6.25..pdf]
